# Supplementary material for: Impact of steroid differentiation on tumor microenvironment revealed by single-nucleus atlas of adrenal tumors
Source: Nat Commun. 2025 Oct 6;16:8860. doi: 10.1038/s41467-025-63912-2 (PMC12501071; doi:10.1038/s41467-025-63912-2)
Supplement: Supplementary file 1 — Supplementary Information [file 41467_2025_63912_MOESM1_ESM.pdf]

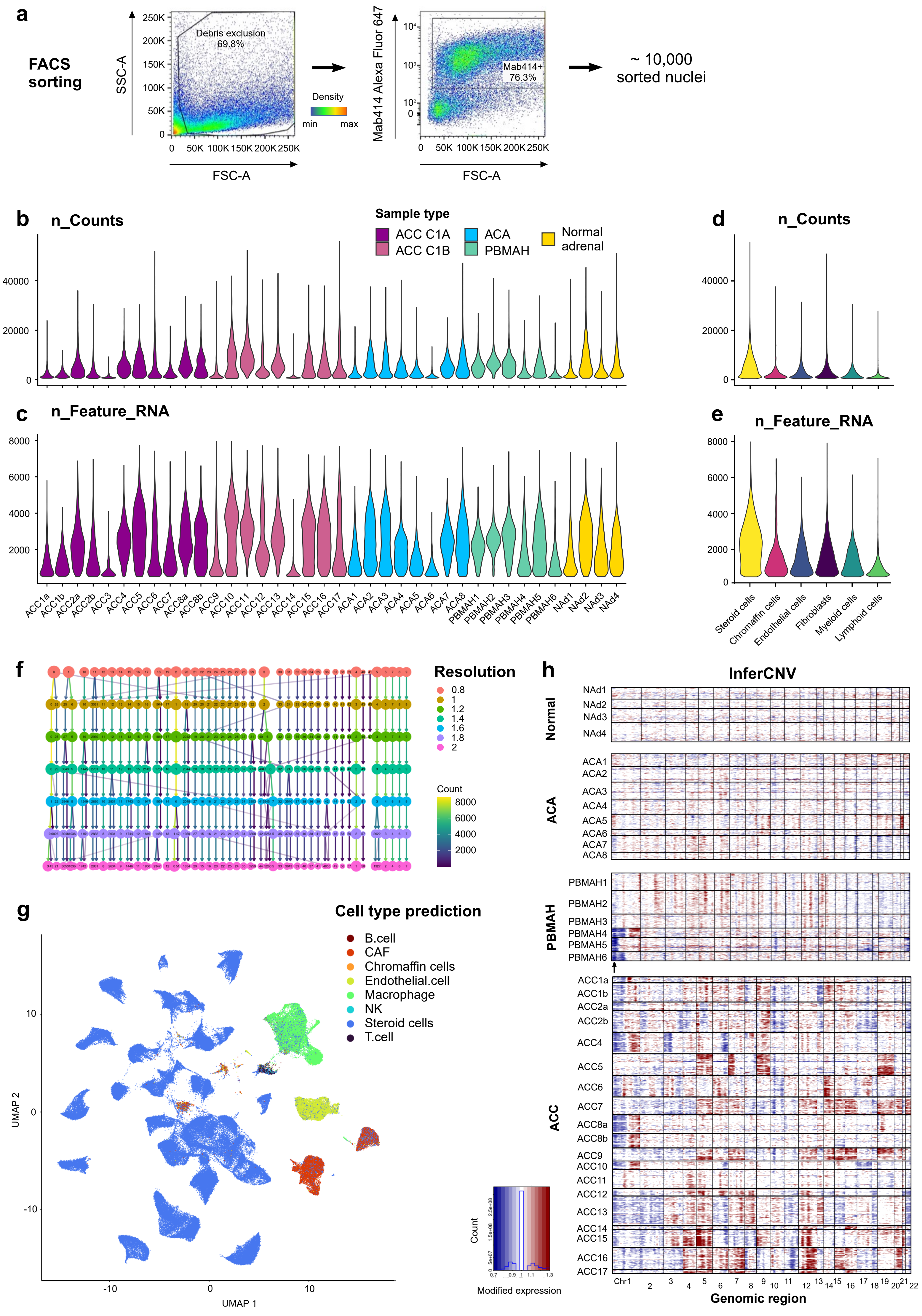

a
FACS sorting
Debris exclusion 69.8%
SSC-A
FSC-A
Density
min
max
Mab414 Alexa Fluor 647
Mab414+ 76.3%
~ 10,000 sorted nuclei
b
n_Counts
Sample type
ACC C1A
ACC C1B
ACA
PBMAH
Normal adrenal
c
n_Feature_RNA
d
n_Counts
e
n_Feature_RNA
Steroid cells
Chromaffin cells
Endothelial cells
Fibroblasts
Myeloid cells
Lymphoid cells
f
Resolution
Count
g
Cell type prediction
B.cell
CAF
Chromaffin cells
Endothelial.cell
Macrophage
NK
Steroid cells
T.cell
UMAP 1
UMAP 2
Modified expression
h
InferCNV
Normal
ACA
PBMAH
ACC
Genomic region

Supplementary Fig. 1. Cell atlas of normal adrenal cortex and adrenocortical tumors: additional representations.

a) Gating strategy for the sorting of nuclei labeled with Alexa Fluor® 647 anti-Nuclear Pore Complex Proteins Antibody (Mab414, BioLegend). The gating strategy consisted of selecting nuclei based on their size and internal complexity on a FSC-A vs. SSC-A dot plot to exclude debris (left panel), and isolating Mab414-positive nuclei on a FSC-A vs. Alexa Fluor® 647 fluorescence dot plot (right panel).

b) and c) Violin plots of unique molecular identifier (UMI) counts (n_Counts) and of distinct transcripts (n_Feature_RNA) in each sample.

d) and e) Violin plots of unique molecular identifier (UMI) counts (n_Counts) and of distinct transcripts (n_Feature_RNA) in each cell type.

f) Clustree representation of clustering stability at different resolutions. Resolution 1.2 was selected.

g) Uniform manifold approximation and projection (UMAP) annotated with cell types predicted using Scibet (for microenvironment) and Garnett (for steroid cells).

h) Chromosome alterations inferred using InferCNV in steroid cells of normal and tumor samples. Predicted gains are colored in red, predicted losses are colored in blue. The black arrow indicates *KDM1A* locus.

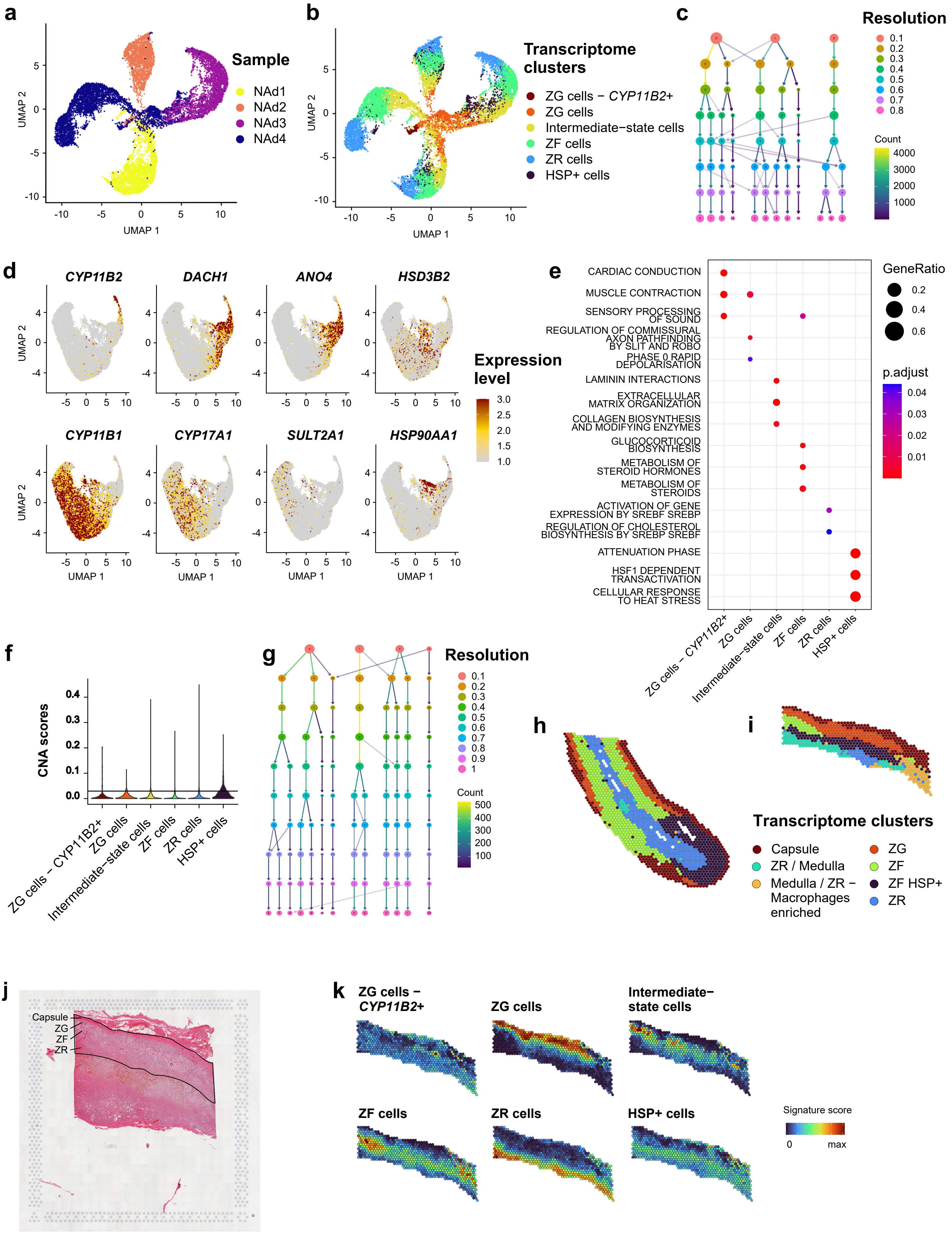
a
Sample
NAd1
NAd2
NAd3
NAd4
UMAP 1
UMAP 2
b
Transcriptome clusters
ZG cells − CYP11B2+
ZG cells
Intermediate−state cells
ZF cells
ZR cells
HSP+ cells
c
Resolution
Count
d
CYP11B2
DACH1
ANO4
HSD3B2
CYP11B1
CYP17A1
SULT2A1
HSP90AA1
Expression level
e
CARDIAC CONDUCTION
MUSCLE CONTRACTION
SENSORY PROCESSING OF SOUND
REGULATION OF COMMISSURAL AXON PATHFINDING BY SLIT AND ROBO
PHASE 0 RAPID DEPOLARISATION
LAMININ INTERACTIONS
EXTRACELLULAR MATRIX ORGANIZATION
COLLAGEN BIOSYNTHESIS AND MODIFYING ENZYMES
GLUCOCORTICOID BIOSYNTHESIS
METABOLISM OF STEROID HORMONES
METABOLISM OF STEROIDS
ACTIVATION OF GENE EXPRESSION BY SREBF SREBP
REGULATION OF CHOLESTEROL BIOSYNTHESIS BY SREBP SREBF
ATTENUATION PHASE
HSF1 DEPENDENT TRANSACTIVATION
CELLULAR RESPONSE TO HEAT STRESS
GeneRatio
p.adjust
f
CNA scores
g
Resolution
Count
h
i
Transcriptome clusters
Capsule
ZR / Medulla
Medulla / ZR − Macrophages enriched
ZG
ZF
ZF HSP+
ZR
j
Capsule
ZG
ZF
ZR
k
ZG cells − CYP11B2+
ZG cells
Intermediate− state cells
ZF cells
ZR cells
HSP+ cells
Signature score
0
max

Supplementary Fig. 2. Characterization of normal steroid cells: additional representations.

a) UMAP annotated by sample identifier ; projection was performed without integration of samples.

b) UMAP annotated by the transcriptome clusters obtained after integration ; projection was performed without integration of samples.

c) Clustree representation of clustering stability at different resolutions, obtained from unsupervised clustering of single-nucleus data. Resolution 0.3 was selected.

d) FeaturePlot of the selected transcriptome cluster markers on UMAP with integration of samples.

e) Gene set enrichment (Reactome) of transcriptome signatures obtained after integration of normal steroid cells.

f) Copy number alterations (CNA) scores inferred from transcriptome. Dashed line represents the threshold (> 3% of genome altered) used to call tumor cells.

g) Clustree representation of clustering stability at different resolutions, obtained from unsupervised clustering of spatial transcriptome data. Resolution 0.2 was selected. Clustering was performed after integration of NAd4 and NAd5, a second normal adrenal sample used for spatial transcriptomics (Seurat *PrepSCTIntegration, FindIntegrationAnchors* and *IntegrateData* functions).

h) Spatial representation of NAd4 (normal adrenal sample), annotated with cluster annotations obtained from unsupervised clustering of spatial transcriptome data.

i) Spatial representation of NAd5, annotated with cluster annotations obtained from unsupervised clustering of spatial transcriptome data.

j) Hematoxylin eosin coloration of NAd5, a second normal adrenal sample used for spatial transcriptomics. Part of the tissue was damaged, only the surrounded part was analyzed.

k) Spatial transcriptomic representation of the 6 signatures of normal steroid cells in NAd5.

Normal steroid cells signatures were deconvoluted with Cell2location in spatial transcriptomics spots.

Abbreviations: ZG, zona glomerulosa ; ZF, zona fasciculata; ZR, zona reticularis ; HSP, heat-shock proteins.

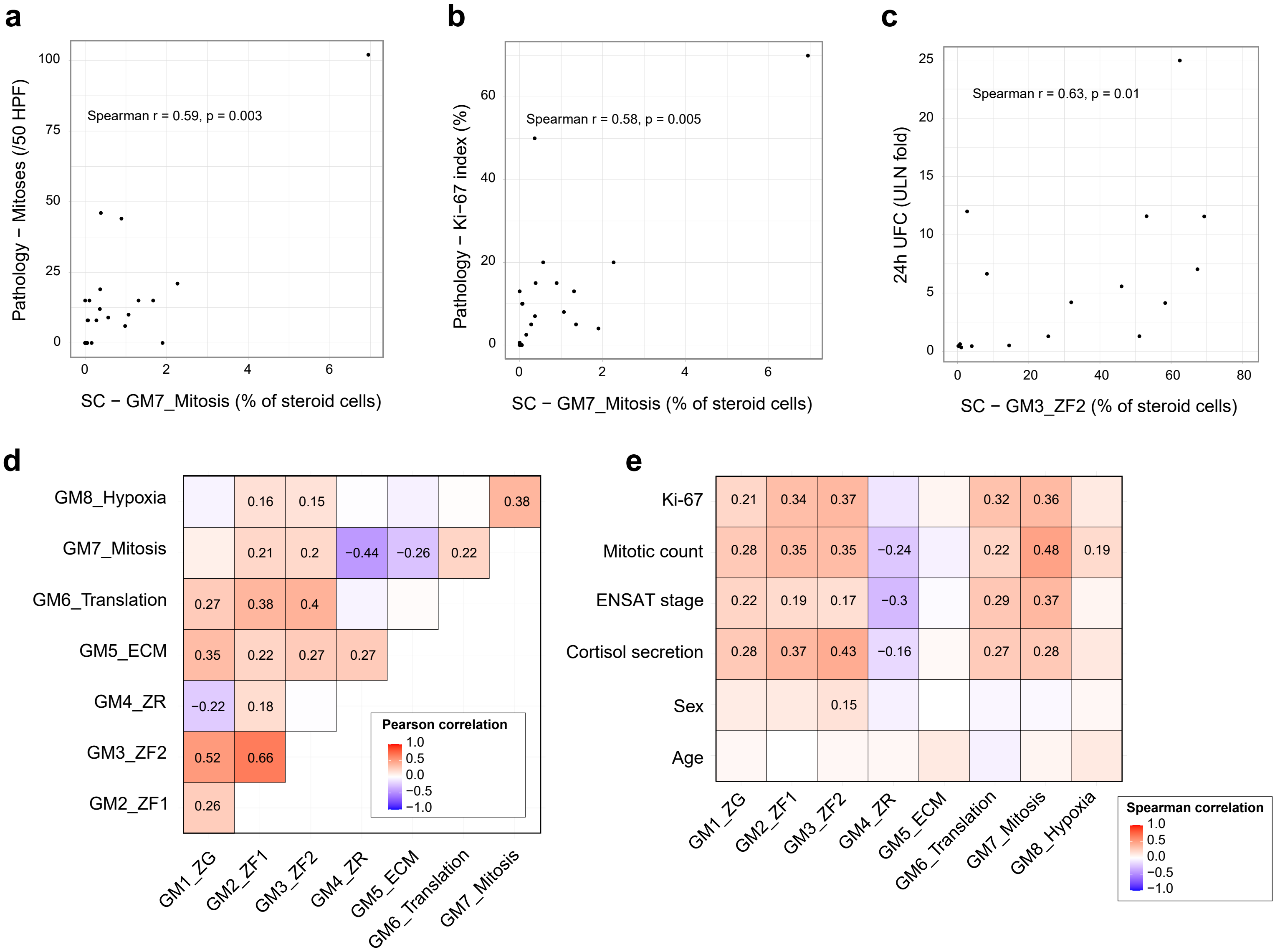

Supplementary Fig. 3. Characterization of tumor steroid cells: additional representations.

a) Correlation (Spearman coefficient) of GM7_Mitosis cells proportion with mitotic count.

b) Correlation (Spearman coefficient) of GM7_Mitosis cells proportion with Ki-67 index.

c) Correlation (Spearman coefficient) of GM3_ZF2 cells proportion with 24h urinary free cortisol (UFC).

d) Correlation (Pearson coefficient) between gene modules scores obtained with ssGSEA in bulk ACC transcriptomes from 201 patients.

e) Correlation (Spearman coefficient) of clinical parameters with gene modules scores obtained with ssGSEA in bulk ACC transcriptomes from 201 patients. Only significant correlations (p-value < 0.05) are displayed.

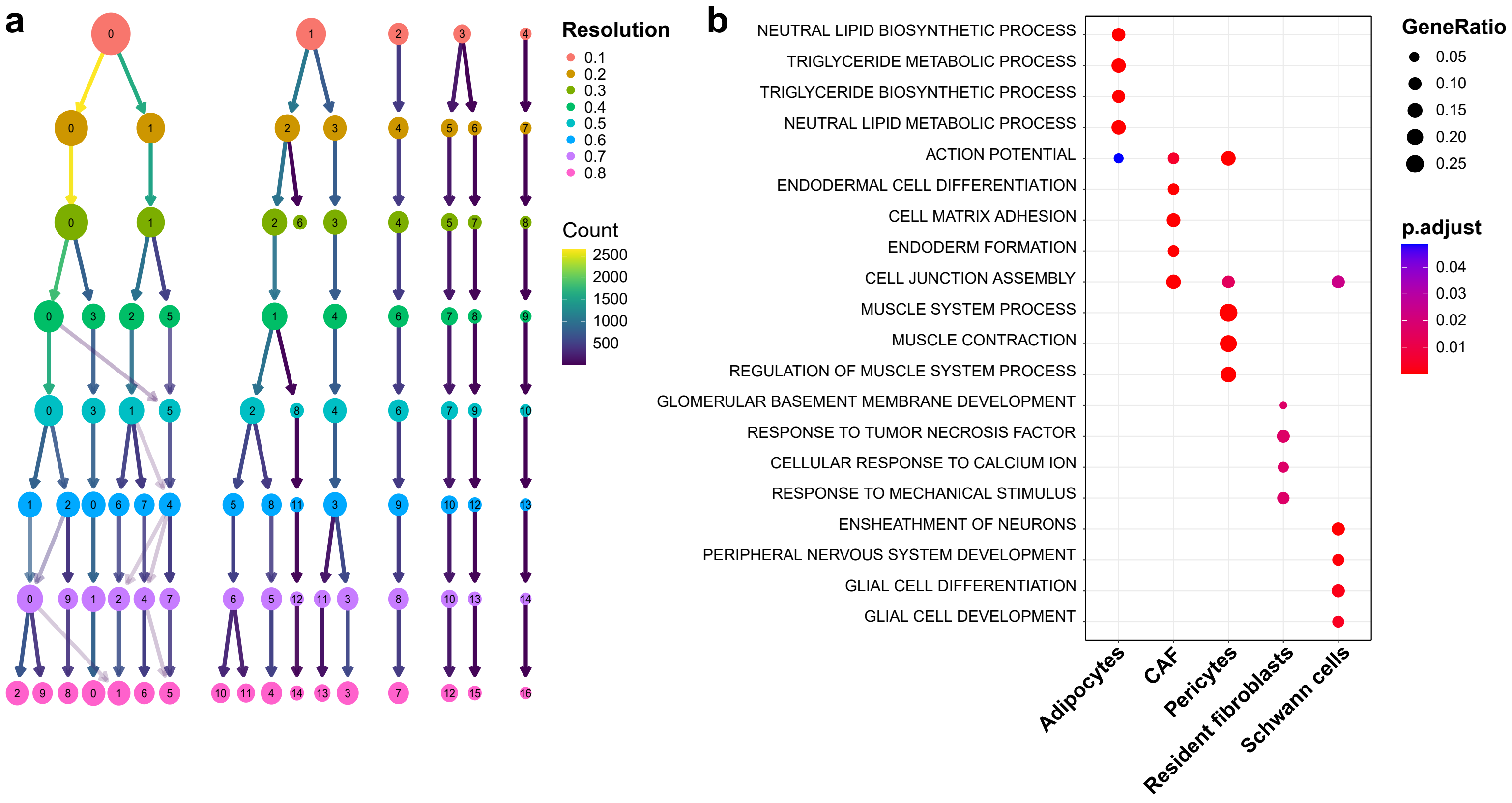

Supplementary Fig. 4. Characterization of adrenocortical fibroblasts: additional representations.
a) Clustree representation of clustering stability at different resolutions. Resolution 0.3 was selected.
b) Gene set enrichment (GO-BP) in CAF and resident fibroblasts.

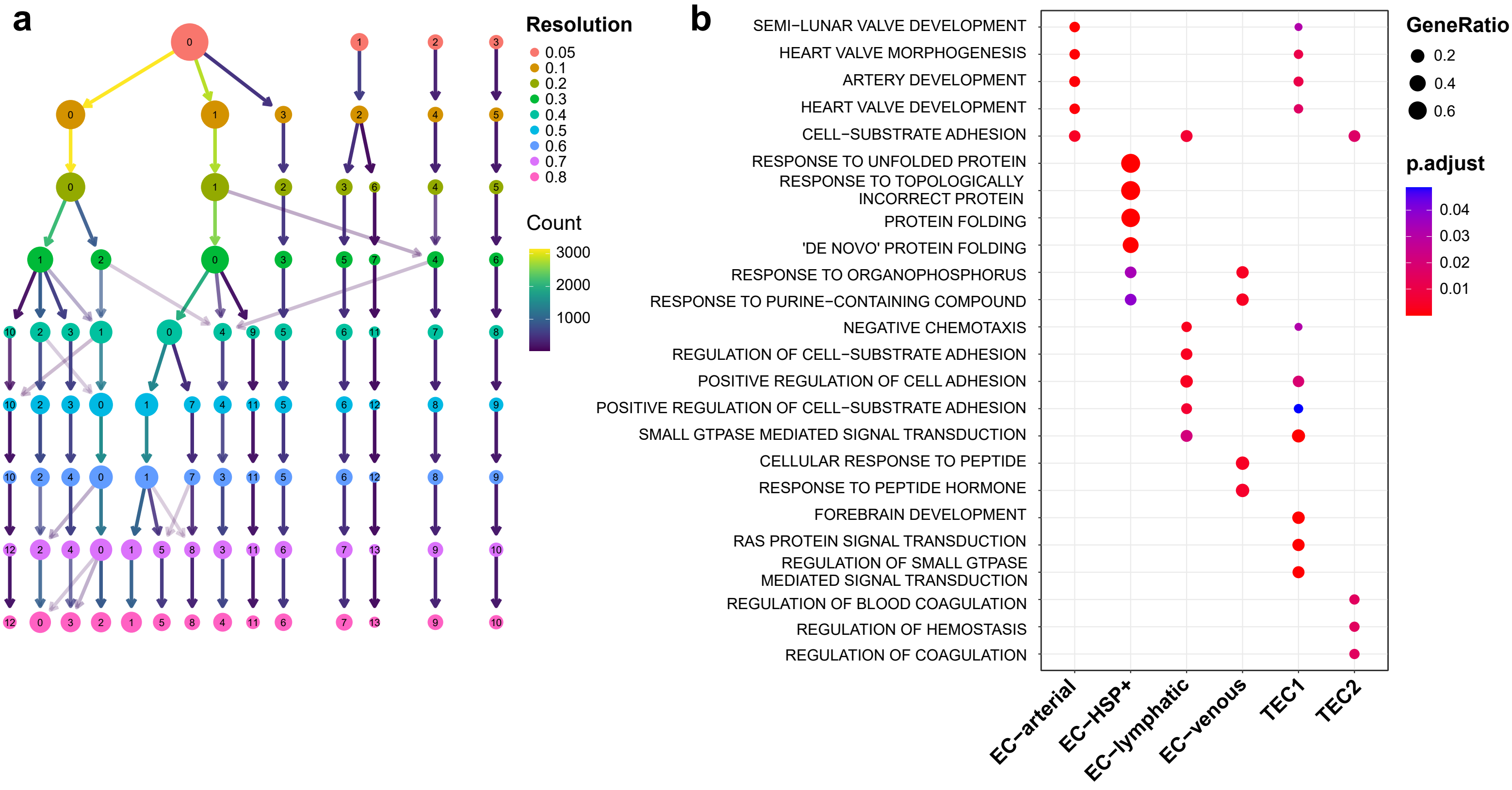

Supplementary Fig. 5. Characterization of adrenocortical endothelial cells: additional representations.

a) Clustree representation of clustering stability at different resolutions. Resolution 0.1 was selected.

b) Gene set enrichment (GO-BP) in adrenocortical endothelial cells.

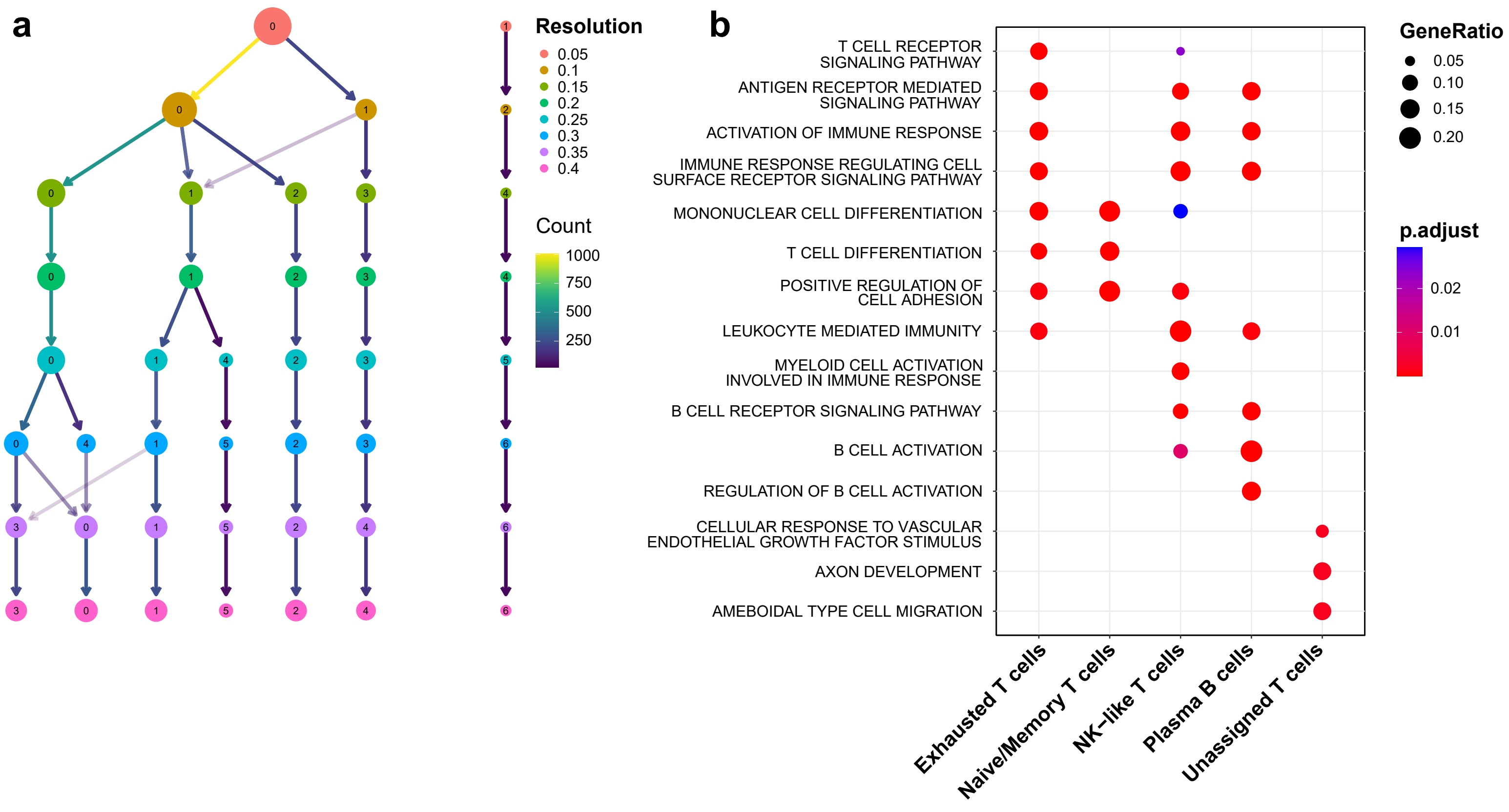

Supplementary Fig. 6. Characterization of adrenocortical lymphocytes: additional representations.
a) Clustree representation of clustering stability at different resolutions. Resolution 0.2 was selected.
b) Gene set enrichment (GO-BP) in adrenocortical lymphocytes.

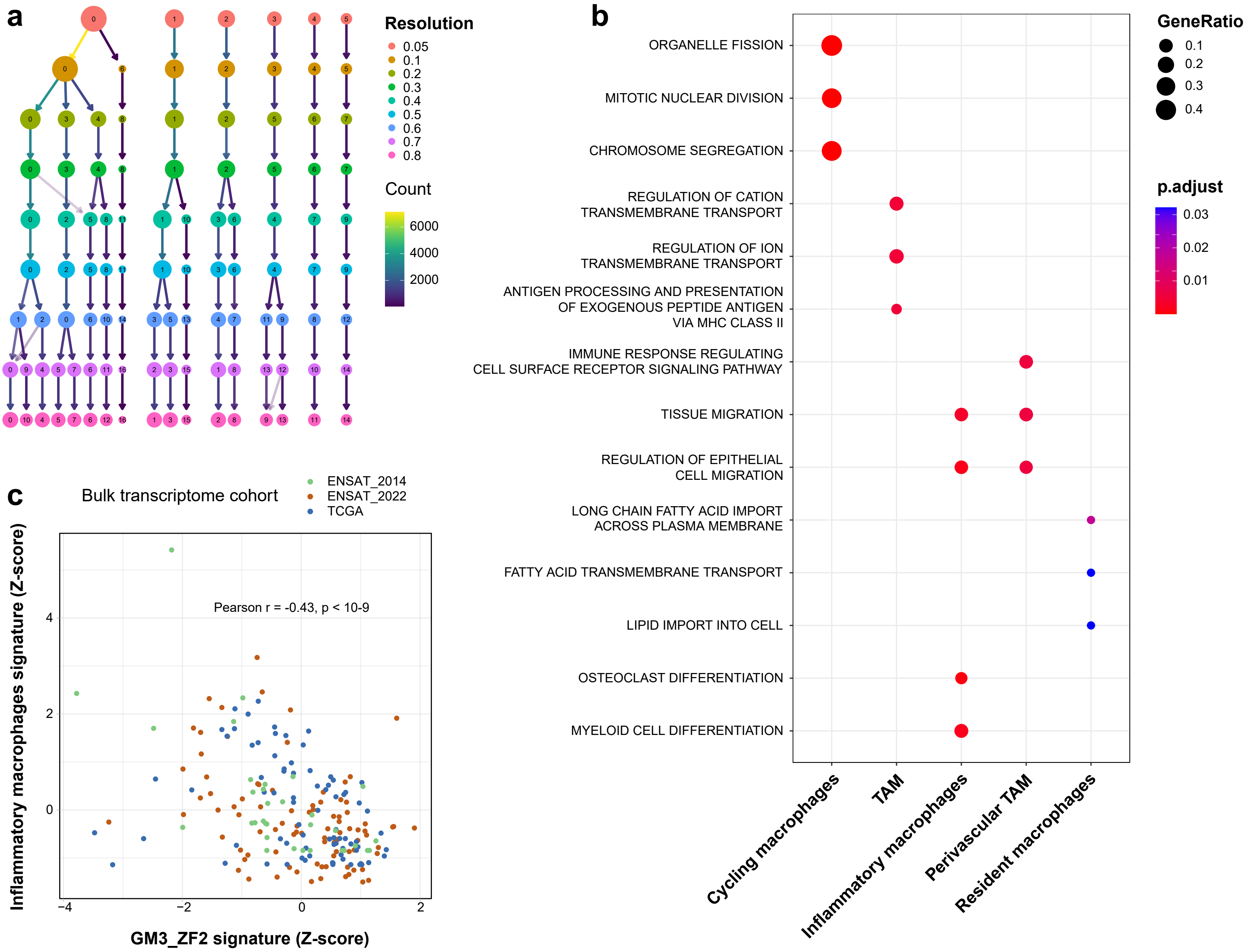

Supplementary Fig. 7. Characterization of adrenocortical myeloid cells: additional representations.
a) Clustree representation of clustering stability at different resolutions. Resolution 0.1 was selected.
b) Gene set enrichment (GO-BP) in adrenocortical myeloid cells.
c) Correlation of inflammatory macrophages and cortisol secretion.
GM3_ZF2 - reflecting cortisol secretion (Suppl Figure 3D and 3F) - was scored with ssGSEA, and inflammatory macrophages signature was deconvoluted with CIBERSORTx in bulk ACC transcriptomes from 201 patients.

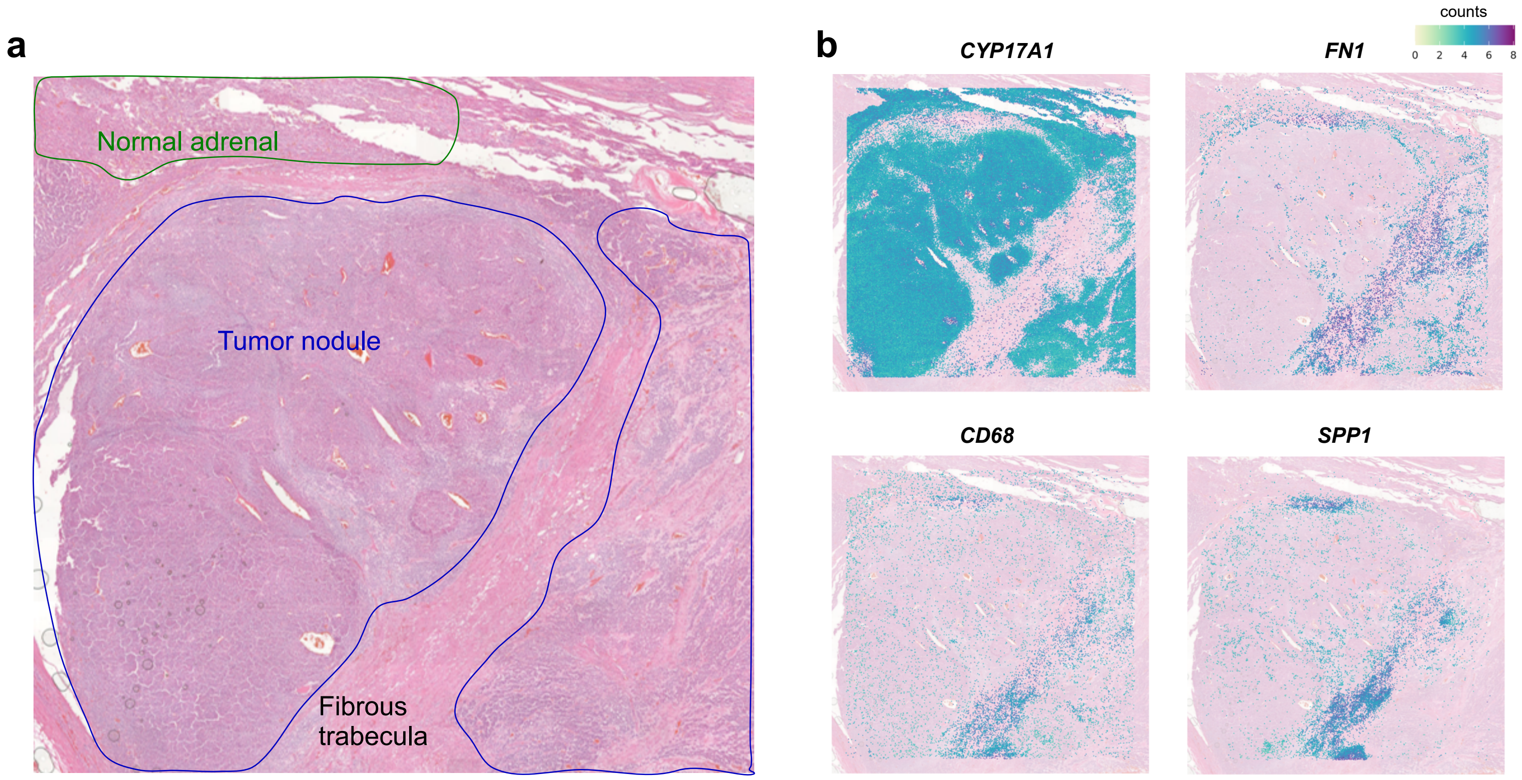

Supplementary Fig. 8. High definition spatial transcriptomics of adrenocortical carcinoma.
a) Hematoxylin eosin coloration of the ACC sample used for high definition spatial transcriptomics.
b) Spatial representation of steroid (*CYP17A1*), fibroblasts (*FN1*) and TAM (*CD68*, *SPP1*) markers.
.

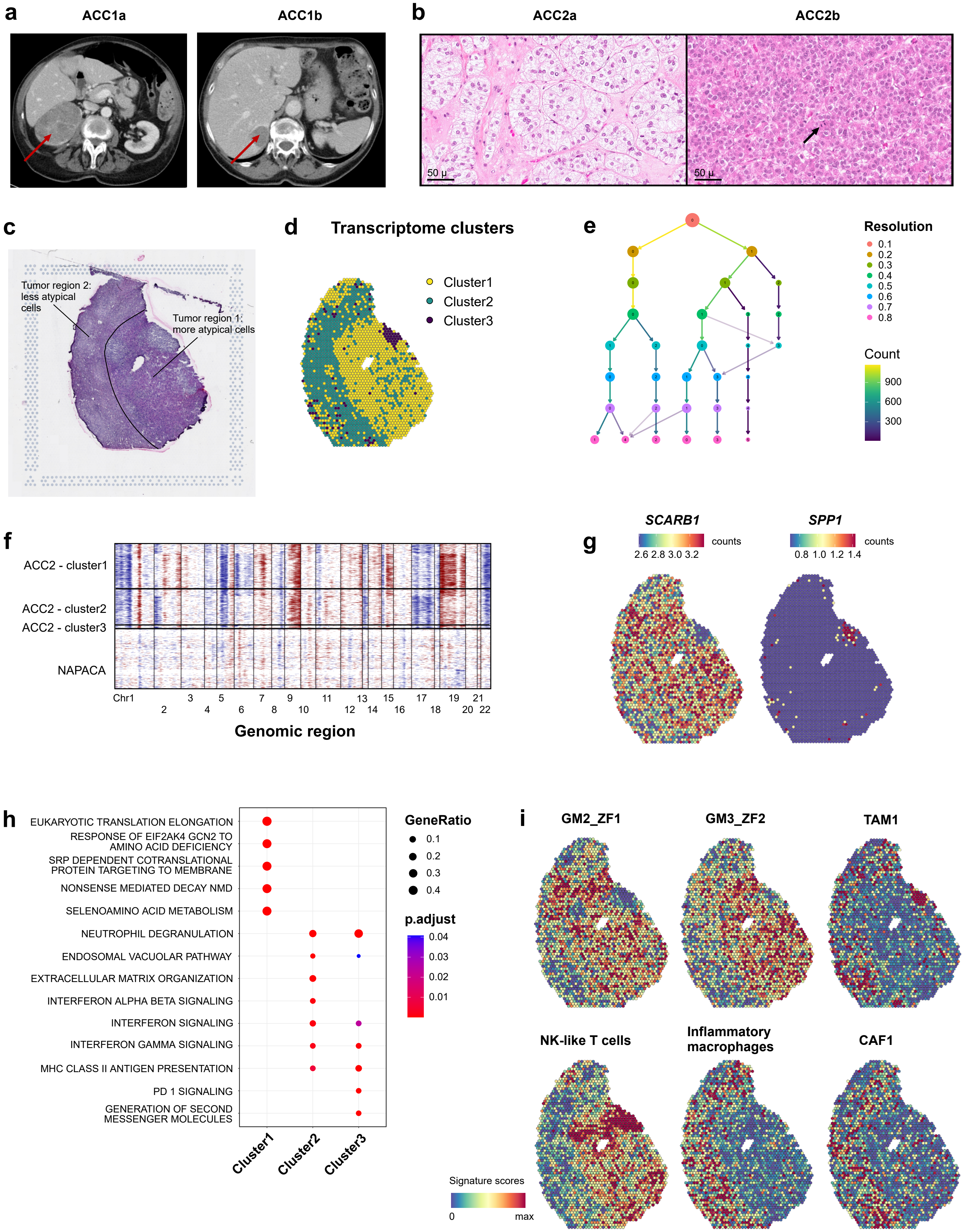
a
ACC1a
ACC1b
b
ACC2a
ACC2b
50 µ
50 µ
c
Tumor region 2:
less atypical
cells
Tumor region 1:
more atypical cells
d
Transcriptome clusters
Cluster1
Cluster2
Cluster3
e
Resolution
0.1
0.2
0.3
0.4
0.5
0.6
0.7
0.8
Count
900
600
300
f
ACC2 - cluster1
ACC2 - cluster2
ACC2 - cluster3
NAPACA
Chr1 2 3 4 5 6 7 8 9 10 11 12 13 14 15 16 17 18 19 20 21 22
Genomic region
g
SCARB1
counts
2.6 2.8 3.0 3.2
SPP1
counts
0.8 1.0 1.2 1.4
h
EUKARYOTIC TRANSLATION ELONGATION
RESPONSE OF EIF2AK4 GCN2 TO AMINO ACID DEFICIENCY
SRP DEPENDENT COTRANSLATIONAL PROTEIN TARGETING TO MEMBRANE
NONSENSE MEDIATED DECAY NMD
SELENOAMINO ACID METABOLISM
NEUTROPHIL DEGRANULATION
ENDOSOMAL VACUOLAR PATHWAY
EXTRACELLULAR MATRIX ORGANIZATION
INTERFERON ALPHA BETA SIGNALING
INTERFERON SIGNALING
INTERFERON GAMMA SIGNALING
MHC CLASS II ANTIGEN PRESENTATION
PD 1 SIGNALING
GENERATION OF SECOND MESSENGER MOLECULES
Cluster1
Cluster2
Cluster3
GeneRatio
0.1
0.2
0.3
0.4
p.adjust
0.04
0.03
0.02
0.01
i
GM2_ZF1
GM3_ZF2
TAM1
NK-like T cells
Inflammatory macrophages
CAF1
Signature scores
0
max

Supplementary Fig. 9. Spatial heterogeneity of single-nucleus signatures.

a) CT-scan images of ACC1. The primary tumor (ACC1a) and metachronous liver metastasis (ACC1b) images are presented.

b) Pathology images of ACC2. One area without aggressive features (ACC2a) and one area with more aggressive features (ACC2b), such as compact eosinophilic cells and high mitotic index (arrowhead), are presented. HES staining, magnification x400, barscale 50 µm.

c) Hematoxylin eosin coloration of the ACC2 sample used for spatial transcriptomics. Cyto-nuclear atypia are more pronounced in tumor region 1.

d) Spatial representation of ACC2, annotated with cluster annotations obtained from unsupervised clustering of spatial transcriptome data.

e) Clustree representation of clustering stability at different resolutions, obtained from unsupervised clustering of spatial transcriptome data. Resolution 0.3 was selected.

f) Chromosome alterations inferred using InferCNV in spatial transcriptomics spots of ACC2 and a benign tumor presented as control. Predicted gains are colored in red, predicted losses are colored in blue.

g) Spatial representation of TAM (*SPP1*) and steroid (*SCARB1*) markers in ACC2.

h) Gene set enrichment (Reactome) in ACC2 spatial transcriptomics.

i) Spatial transcriptomic representation of 2 gene modules scores (GM2_ZF1, GM3_ZF2) and 4 microenvironment transcriptome signatures (TAM1, Inflammatory macrophages, NK-like T-cells, CAF1) in ACC2.

Gene modules scores were obtained using the *AddModuleScore* Seurat function. Microenvironment signatures were obtained using Cell2Location deconvolution.

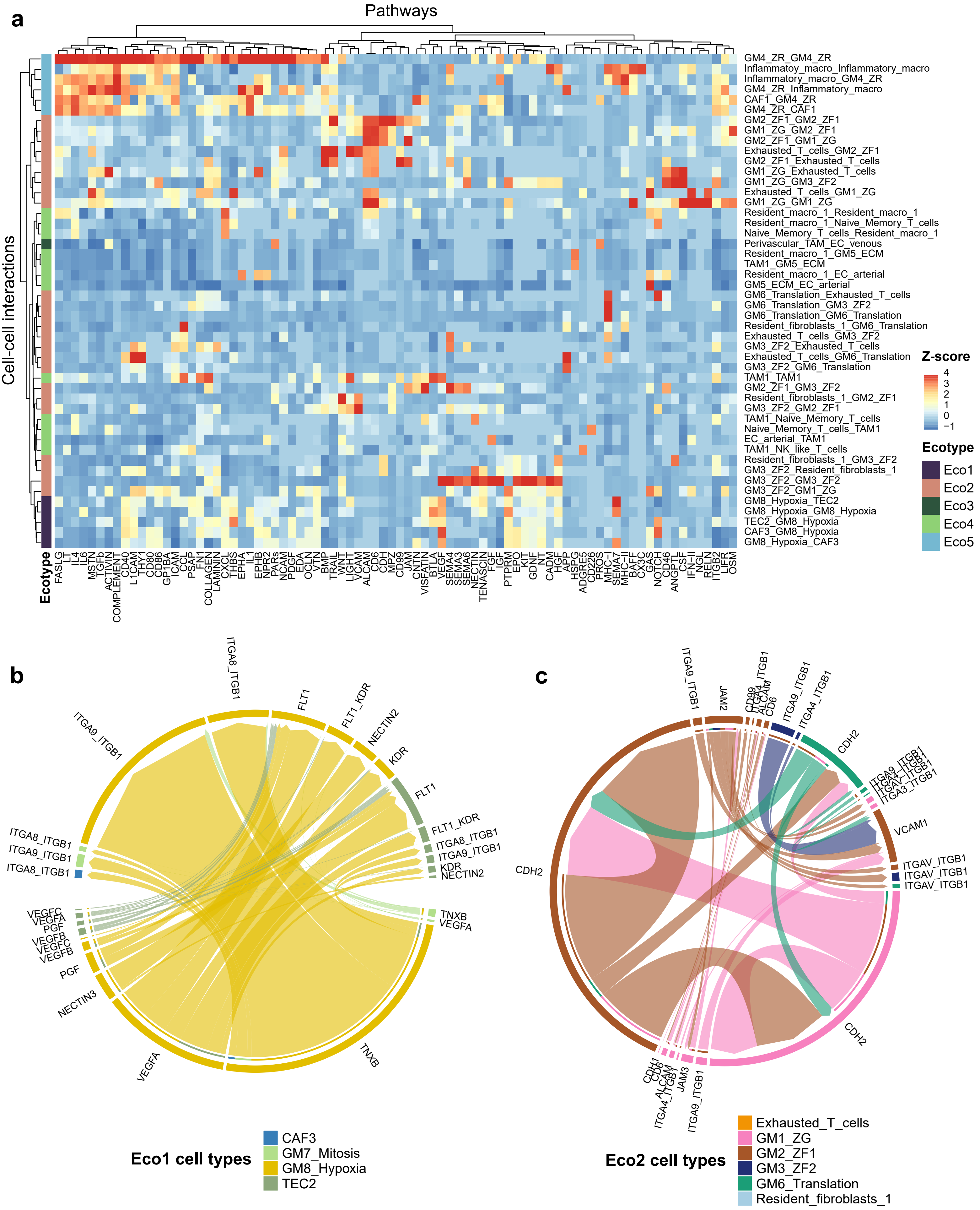
a
Pathways
Cell-cell interactions
Z-score
Ecotype
Eco1
Eco2
Eco3
Eco4
Eco5
b
Eco1 cell types
CAF3
GM7_Mitosis
GM8_Hypoxia
TEC2
c
Eco2 cell types
Exhausted_T_cells
GM1_ZG
GM2_ZF1
GM3_ZF2
GM6_Translation
Resident_fibroblasts_1

Supplementary Fig. 10. Interactions between single-nucleus signatures: additional representations.

a) Hierarchical clustering of the main cell-cell interaction pathways in single-nucleus transcriptomes.

Probabilities of cell-cell interactions were computed for all possible ligand-receptor pairs using Cellchat, then integrated into signaling pathway interaction scores. The heatmap of pathway interaction scores (Z-scores) is presented. Ligand-receptor pairs related to angiogenesis, cell adhesion and inflammation pathways are enriched in Eco1, Eco2 and Eco5 respectively.

b) Chord plot representation of main ligand-receptor interactions related to angiogenesis in Eco1 ecotype.

Ligand-receptor pairs interactions related to angiogenesis are presented for the following signaling pathways enriched in Eco1: VEGF, NECTIN, TENASCIN, EPO and HGF.

c) Chord plot representation of main ligand-receptor interactions related to cell adhesion in Eco2 ecotype.

Ligand-receptor pairs interactions related to cell adhesion are presented for the following signaling pathways enriched in Eco1: VCAM, ALCAM, CD6, CDH, CD99 and JAM.
