# Supplementary material for: Impact of steroid differentiation on tumor microenvironment revealed by single-nucleus atlas of adrenal tumors
Source: Nat Commun. 2025 Oct 6;16:8860. doi: 10.1038/s41467-025-63912-2 (PMC12501071; doi:10.1038/s41467-025-63912-2)
Supplement: Supplementary file 3 — Description of Additional Supplementary Files [file 41467_2025_63912_MOESM3_ESM.pdf]

Description of Additional Supplementary Files

Supplementary Data 1. Samples details.
*germline alteration

Supplementary Data 2. Patients characteristics.
Median (IQR) or number (%)

Supplementary Data 3. Cell-type signatures in normal adrenal cortex and adrenocortical tumors.
Top 100 most differentially expressed genes (Wilcoxon test, Benjamini-Hochberg adjustment) between cell types are presented.

Supplementary Data 4. Transcriptome cluster signatures in normal steroid cells.
Top 100 most differentially expressed genes (Wilcoxon test, Benjamini-Hochberg adjustment) between transcriptome clusters are presented.

Supplementary Data 5. Gene expression associated with pseudotime in normal steroid cells.
For each gene associated with pseudotime, the following variables are provided: p- (permutation test) and q-value (Benjamini-Hochberg adjustment) for association with pseudotime, and mean gene expression (raw-scaled counts) in transcriptome clusters of normal steroid cells.

Supplementary Data 6. Transcriptome cluster signatures in spatial transcriptomics of normal adrenal samples.
Clusters were obtained from unsupervised clustering of spatial transcriptome data after integration of NAd4 and NAd5 (Seurat PrepSCTIntegration, FindIntegrationAnchors and IntegrateData functions).
Top 100 most differentially expressed genes (Wilcoxon test, Benjamini-Hochberg adjustment) between transcriptome clusters are presented.

Supplementary Data 7. Recurrent gene modules in tumor steroid cells.

Supplementary Data 8. Disease-free and overall survival models including steroid and microenvironment signatures.
Using univariate and stepwise multivariate Cox models in 201 patients with ACC, the following variables were tested:
- Gene modules scores obtained with ssGSEA in bulk transcriptomes
- Microenvironement transcriptome signatures deconvoluted with CIBERSORTx in bulk transcriptomes
- Clinical variables with known prognostic value (ENSAT stage, cortisol secretion, Ki-67, mitotic count).

Supplementary Data 9. Transcriptome cluster signatures in adrenocortical fibroblasts.
Top 100 most differentially expressed genes (Wilcoxon test, Benjamini-Hochberg adjustment) between cell types are presented.

Supplementary Data 10. Gene expression associated with pseudotime in adrenocortical fibroblasts.
For each gene associated with pseudotime, the following variables are provided: p- (permutation test) and q-value (Benjamini-Hochberg adjustment) for association with branched pseudotime, and mean gene expression (raw-scaled counts) in transcriptome clusters of adrenocortical fibroblasts.

Supplementary Data 11. Transcriptome cluster signatures in adrenocortical endothelial cells.
Top 100 most differentially expressed genes (Wilcoxon test, Benjamini-Hochberg adjustment) between cell types are presented.

Supplementary Data 12. Gene expression associated with pseudotime in adrenocortical endothelial cells.
For each gene associated with pseudotime, the following variables are provided: p- (permutation test) and q-value (Benjamini-Hochberg adjustment) for association with branched pseudotime, and mean gene expression (raw-scaled counts) in transcriptome clusters of adrenocortical endothelial cells.

Supplementary Data 13. Transcriptome cluster signatures in adrenocortical lymphocytes.
Top 100 most differentially expressed genes (Wilcoxon test, Benjamini-Hochberg adjustment) between cell types are presented.

Supplementary Data 14. Gene expression associated with pseudotime in adrenocortical lymphocytes.
For each gene associated with pseudotime, the following variables are provided: p- (permutation test) and q-value (Benjamini-Hochberg adjustment) for association with branched pseudotime, and mean gene expression (raw-scaled counts) in transcriptome clusters of adrenocortical lymphocytes.

Supplementary Data 15. Transcriptome cluster signatures in adrenocortical myeloid cells.
Top 100 most differentially expressed genes (Wilcoxon test, Benjamini-Hochberg adjustment) between cell types are presented.

Supplementary Data 16. Gene expression associated with pseudotime in adrenocortical myeloid cells.
For each gene associated with pseudotime, the following variables are provided: p- (permutation test) and q-value (Benjamini-Hochberg adjustment) for association with branched pseudotime, and mean gene expression (raw-scaled counts) in transcriptome clusters of adrenocortical myeloid cells.

Supplementary Data 17. Intra-tumor heterogeneity of cell composition and of single- nucleus signatures.
P-values are computed using Fisher's test with a Monte Carlo simulation approach, with 2000 replicates.

Supplementary Data 18. Transcriptome cluster signatures in spatial transcriptomics of ACC2.
Top 100 most differentially expressed genes (Wilcoxon test, Benjamini-Hochberg adjustment) between transcriptome clusters are presented.

Supplementary Data 19. Ligand-receptor pair interactions in ecotypes.
P-values are computed with one-sided permutation test.

Supplementary Data 20. Disease-free and overall survival models including ENSAT stage and ecotypes.
Univariate and stepwise multivariate Cox models were performed in 201 patients with ACC.

Supplementary Data 21. C-indexes of disease-free and overall survival models.
Cox models were performed in 201 patients with ACC.
Combined model including clinical variables and ecotypes reached higher C-indexes for predicting DFS (LRT $p = 1.5e-05$) and OS (LRT $p = 0.0005$) than clinical variables alone.
LRT, likelihood ratio test.
