# Supplementary material for: Impact of steroid differentiation on tumor microenvironment revealed by single-nucleus atlas of adrenal tumors
Source: Nat Commun. 2025 Oct 6;16:8860. doi: 10.1038/s41467-025-63912-2 (PMC12501071; doi:10.1038/s41467-025-63912-2)
Supplement: Supplementary file 5 — Reporting Summary [file 41467_2025_63912_MOESM5_ESM.pdf]

nature portfolio

Corresponding author(s): Guillaume Assié, Anne Jouinot

Last updated by author(s): Jul 18, 2025

Reporting Summary

Nature Portfolio wishes to improve the reproducibility of the work that we publish. This form provides structure for consistency and transparency in reporting. For further information on Nature Portfolio policies, see our Editorial Policies and the Editorial Policy Checklist.

Statistics

For all statistical analyses, confirm that the following items are present in the figure legend, table legend, main text, or Methods section.

n/a	Confirmed	
☐	☒	The exact sample size (*n*) for each experimental group/condition, given as a discrete number and unit of measurement
☐	☒	A statement on whether measurements were taken from distinct samples or whether the same sample was measured repeatedly
☐	☒	The statistical test(s) used AND whether they are one- or two-sided<br>*Only common tests should be described solely by name; describe more complex techniques in the Methods section.*
☐	☒	A description of all covariates tested
☐	☒	A description of any assumptions or corrections, such as tests of normality and adjustment for multiple comparisons
☐	☒	A full description of the statistical parameters including central tendency (e.g. means) or other basic estimates (e.g. regression coefficient) AND variation (e.g. standard deviation) or associated estimates of uncertainty (e.g. confidence intervals)
☐	☒	For null hypothesis testing, the test statistic (e.g. *F*, *t*, *r*) with confidence intervals, effect sizes, degrees of freedom and *P* value noted<br>*Give P values as exact values whenever suitable.*
☒	☐	For Bayesian analysis, information on the choice of priors and Markov chain Monte Carlo settings
☒	☐	For hierarchical and complex designs, identification of the appropriate level for tests and full reporting of outcomes
☐	☒	Estimates of effect sizes (e.g. Cohen's *d*, Pearson's *r*), indicating how they were calculated

Our web collection on statistics for biologists contains articles on many of the points above.

Software and code

Policy information about availability of computer code

Data collection	No specific software was used for data collection of public datasets.<br>The public transcriptome datasets used in this study are openly available and can be accessed as follows:<br>- “ENSAT_2014” in the Gene Expression Omnibus (GEO) repository under accession number GSE49280 [https://www.ncbi.nlm.nih.gov/geo/query/acc.cgi?acc=GSE49280].<br>- “TCGA_2016” in the GDC portal [https://portal.gdc.cancer.gov/projects/TCGA-ACC]<br>- “ENSAT_2022” in the supplementary tables of the original paper [https://academic.oup.com/ejendo/article/186/6/607/6853696#supplementary-data]
Data analysis	Data analysis was performed using R (v.4.3.1) using the following packages: Seurat v4.3.0, Clustree v0.5.0, SciBet c1.0, Garnett c0.1.23, InferCNV v1.18.1, ClusterProfiler v4.10.0, Monocle v2.30.0, GSVA v1.36.2, cola v2.8.0, and CellChat v1.6.1.<br>Additional software included Cell Ranger (v3.1.0, 10x Genomics), Space Ranger (v1.3.1, 10x Genomics), Scrublet v0.2.3, CIBERSORTx (Docker version) and Cell2location v0.1.3.<br>Code related to the analyses in this study can be found on Github https://github.com/GESTE-IC/snRNAseq_ACT_atlas.

For manuscripts utilizing custom algorithms or software that are central to the research but not yet described in published literature, software must be made available to editors and reviewers. We strongly encourage code deposition in a community repository (e.g. GitHub). See the Nature Portfolio guidelines for submitting code & software for further information.

Data

Policy information about availability of data

All manuscripts must include a data availability statement. This statement should provide the following information, where applicable:

- Accession codes, unique identifiers, or web links for publicly available datasets
- A description of any restrictions on data availability
- For clinical datasets or third party data, please ensure that the statement adheres to our policy

The raw single-nucleus RNA-sequencing and spatial transcriptomics data generated in this study have been anonymized using BAMboozle78 and deposited in the European Genome-phenome Archive (EGA) database under accession code EGAD50000000835.
Counts tables of single-nucleus RNA-sequencing have been deposited on EGA under accession code EGAD50000000836 and on Zenodo79 [10.5281/zenodo.10534061] (ACC) and [10.5281/zenodo.10534245] (benign ACT and normal adrenals).
Counts tables and images of spatial transcriptomics have been deposited on EGA under accession code EGAD50000000836 and on Zenodo79 [10.5281/zenodo.10560206] (ACC) and [10.5281/zenodo.10560525] (normal adrenals).

The public transcriptome datasets used in this study are openly available and can be accessed as follows:
- "ENSAT_2014" in the Gene Expression Omnibus (GEO) repository under accession number GSE49280 [https://www.ncbi.nlm.nih.gov/geo/query/acc.cgi?acc=GSE49280].
- "TCGA_2016" in the GDC portal [https://portal.gdc.cancer.gov/projects/TCGA-ACC]
- "ENSAT_2022" in the supplementary tables of the original paper22 [https://academic.oup.com/ejendo/article/186/6/607/6853696#supplementary-data]

Research involving human participants, their data, or biological material

Policy information about studies with human participants or human data. See also policy information about sex, gender (identity/presentation), and sexual orientation and race, ethnicity and racism.

Reporting on sex and gender	Findings apply to all sex and gender. Gender was determined based on self-report and was considered in the study design to reflect the sex ratios typically observed in the different types of adrenal tumors.<br>The study included 29 females / 7 males, which represents an usual sex-ratio in adrenocortical tumors.<br>Signed informed consent for access to clinical data was obtained from each patient. Agregated patient's details including gender are reported in Suppl Table 2.<br>Sex or gender were not included in analyses, as they were neither associated with transcriptome clusters, nor considered as prognostic factors in the field of adrenal tumors.
Reporting on race, ethnicity, or other socially relevant groupings	This study does not report race, ethinicity or other socially relevant groupings.
Population characteristics	36 adult (median age 45y, range 28-77) patients and 39 tissue samples (20 adrenocortical carcinomas, 8 adrenocortical adenomas, 6 primary bilateral macronodular hyperplasias, 5 normal adrenals) were included in the study.
Recruitment	Patients who consented to the study and fit one histological or molecular group of interest, with available and good quality frozen tissue samples, were recruited for single-nuclei RNA-sequencing. Our study had no other biases in the recruitement of patients.
Ethics oversight	This study was reviewed and approved by the Institutional Review Board "Comité de protection des personnes Ile de France 1" (application #13311 COMETE-TACTIC). Signed informed consent for somatic molecular analysis and for access to de-identified clinical data was obtained from each patient.

Note that full information on the approval of the study protocol must also be provided in the manuscript.

Field-specific reporting

Please select the one below that is the best fit for your research. If you are not sure, read the appropriate sections before making your selection.

☒ Life sciences ☐ Behavioural & social sciences ☐ Ecological, evolutionary & environmental sciences

For a reference copy of the document with all sections, see nature.com/documents/nr-reporting-summary-flat.pdf

Life sciences study design

All studies must disclose on these points even when the disclosure is negative.

Sample size	All samples sizes are indicated in the manuscript. No sample size calculation was performed, due to the exploratory nature of the study.<br>The samples analyzed in this study reflect the main adrenocortical tumor types, with a minimum of 3 samples per tumor group.

Data exclusions	Doublets from single-nuclei RNA-sequencing were filtered out using Scrublet. In addition, low-quality nuclei were filtered out by removing nuclei with < 500 detected genes, > 8,000 detected genes, or > 5% of mitochondrial transcripts.<br>For spatial transcriptomics data, all spatial barcodes that did not fall under tissue were removed.
Replication	As it is typical in this field, single-nuclei RNA-sequencing and spatial transcriptomics data were not replicated due to limited tissue samples and cost of experiments.<br>The findings of the study were validated using deconvolution in bulk transcriptomes of 3 independent cohorts (TCGA_2016, ENSAT_2014, ENSAT_2022).
Randomization	Considering the exploratory nature of the study, randomization was not relevant.
Blinding	Investigators were not blinded to tumor group, since this information was required to select the samples for the study, and to design and guide analyses of single-nuclei RNA-sequencing and spatial transcriptomics data.

Reporting for specific materials, systems and methods

We require information from authors about some types of materials, experimental systems and methods used in many studies. Here, indicate whether each material, system or method listed is relevant to your study. If you are not sure if a list item applies to your research, read the appropriate section before selecting a response.

Materials & experimental systems

n/a	Involved in the study
☐	☒ Antibodies
☒	☐ Eukaryotic cell lines
☒	☐ Palaeontology and archaeology
☒	☐ Animals and other organisms
☒	☐ Clinical data
☒	☐ Dual use research of concern
☒	☐ Plants

Methods

n/a	Involved in the study
☒	☐ ChIP-seq
☒	☐ Flow cytometry
☒	☐ MRI-based neuroimaging

Antibodies

Antibodies used	Alexa Fluor® 647 anti-Nuclear Pore Complex Proteins Antibody clone Mab414, 1:250, RRID:AB_2728507, Biolegend, cat number 682203<br>anti-DAB2 clone HPA028888, 1:800, Sigma Aldricht<br>anti-CYP17A1 clone HPA048533, 1:800, Sigma Aldricht<br>anti-SPP1 clone HPA027541, 1: 475, Sigma Aldricht
Validation	These commercial antibodies were acquired from Biolegend (anti-Nuclear Pore) and Sigma-Aldrich (anti-DAB2, CYP17A1 and SPP1) and validated by the suppliers. Antibodies validation can be found on manufacturer websites (https://www.biolegend.com/ and https://www.sigmaaldrich.com/).

Plants

Seed stocks	*Report on the source of all seed stocks or other plant material used. If applicable, state the seed stock centre and catalogue number. If plant specimens were collected from the field, describe the collection location, date and sampling procedures.*
Novel plant genotypes	*Describe the methods by which all novel plant genotypes were produced. This includes those generated by transgenic approaches, gene editing, chemical/radiation-based mutagenesis and hybridization. For transgenic lines, describe the transformation method, the number of independent lines analyzed and the generation upon which experiments were performed. For gene-edited lines, describe the editor used, the endogenous sequence targeted for editing, the targeting guide RNA sequence (if applicable) and how the editor was applied.*
Authentication	*Describe any authentication procedures for each seed stock used or novel genotype generated. Describe any experiments used to assess the effect of a mutation and, where applicable, how potential secondary effects (e.g. second site T-DNA insertions, mosiacism, off-target gene editing) were examined.*
